# Supplementary material for: Pericyte remodeling is deficient in the aged brain and contributes to impaired capillary flow and structure
Source: Nat Commun. 2022 Oct 7;13:5912. doi: 10.1038/s41467-022-33464-w (PMC9547063; doi:10.1038/s41467-022-33464-w)
Supplement: Supplementary file 3 — Reporting Summary [file 41467_2022_33464_MOESM3_ESM.pdf]

nature portfolio

Corresponding author(s): Andy Y. Shih
Last updated by author(s): 01/09/2022

Reporting Summary

Nature Portfolio wishes to improve the reproducibility of the work that we publish. This form provides structure for consistency and transparency in reporting. For further information on Nature Portfolio policies, see our Editorial Policies and the Editorial Policy Checklist.

Statistics

For all statistical analyses, confirm that the following items are present in the figure legend, table legend, main text, or Methods section.

n/a	Confirmed	
☐	☒	The exact sample size (n) for each experimental group/condition, given as a discrete number and unit of measurement
☐	☒	A statement on whether measurements were taken from distinct samples or whether the same sample was measured repeatedly
☐	☒	The statistical test(s) used AND whether they are one- or two-sided<br>*Only common tests should be described solely by name; describe more complex techniques in the Methods section.*
☐	☒	A description of all covariates tested
☐	☒	A description of any assumptions or corrections, such as tests of normality and adjustment for multiple comparisons
☐	☒	A full description of the statistical parameters including central tendency (e.g. means) or other basic estimates (e.g. regression coefficient) AND variation (e.g. standard deviation) or associated estimates of uncertainty (e.g. confidence intervals)
☐	☒	For null hypothesis testing, the test statistic (e.g. F, t, r) with confidence intervals, effect sizes, degrees of freedom and P value noted<br>*Give P values as exact values whenever suitable.*
☒	☐	For Bayesian analysis, information on the choice of priors and Markov chain Monte Carlo settings
☐	☒	For hierarchical and complex designs, identification of the appropriate level for tests and full reporting of outcomes
☒	☐	Estimates of effect sizes (e.g. Cohen's d, Pearson's r), indicating how they were calculated

Our web collection on statistics for biologists contains articles on many of the points above.

Software and code

Policy information about availability of computer code

Data collection	In vivo imaging data was collected using PrairieView software (version 5.5) for the Bruker Investigator multi-photon microscope.
Data analysis	Analysis of in vivo imaging data was performed with GraphPad Prism (version 9), ImageJ (version 2.1.0), Imaris x64 (version 7.7.2), and MATLAB (R2021a) software. Analysis of in silico data was performed using Python (version 2.7) and 3D-visualizations (Figure 8a-c) were performed with the open source software Paraview (v5.7.0). The in silico data generated for this study together with analyses scripts and instructions have been deposited at https://doi.org/10.5281/zenodo.7038939. Further details and explanations of in silico data are available from F.S. upon request.

For manuscripts utilizing custom algorithms or software that are central to the research but not yet described in published literature, software must be made available to editors and reviewers. We strongly encourage code deposition in a community repository (e.g. GitHub). See the Nature Portfolio guidelines for submitting code & software for further information.

Data

Policy information about availability of data

All manuscripts must include a data availability statement. This statement should provide the following information, where applicable:
- Accession codes, unique identifiers, or web links for publicly available datasets
- A description of any restrictions on data availability
- For clinical datasets or third party data, please ensure that the statement adheres to our policy

Source data are provided with this paper. Raw image files are stored on servers at Seattle Children’s Research Institute owing to their large size. These raw data can be provided by the corresponding author upon request.

Field-specific reporting

Please select the one below that is the best fit for your research. If you are not sure, read the appropriate sections before making your selection.

- [x] Life sciences
- [ ] Behavioural & social sciences
- [ ] Ecological, evolutionary & environmental sciences

For a reference copy of the document with all sections, see nature.com/documents/nr-reporting-summary-flat.pdf

Life sciences study design

All studies must disclose on these points even when the disclosure is negative.

Sample size	We did not use statistical methods to predetermine sample size. Sample sizes in this study were estimated based on our prior studies that have used n=6 mice per experimental group to measure differences in capillary diameter or flow in response to optical manipulation of pericytes (PMID: 33603231).
Data exclusions	Data was excluded when there was immediate laser-induced injury to the capillary wall during optical ablation of pericyte or off-target sham irradiations.
Replication	The number of replicates per experiment (and number of mice used per experiment) are noted in the relevant figure legends with comparison of adult versus aged mice. For representative images, we state in the figure legend how often the experiment was repeated independently with similar results.
Randomization	No formal randomization of mice was performed for this study since there was no drug or treatment comparisons within the same age group. However, both sexes were used and mice were derived from different litters and different breeder pairs within the colony. Specifically, litters of PDGFRβ-tdTomato mice (both males and females derived from breeding of male PDGFRβ-Cre and female Ai14 mice) were held for aging until an appropriate time for experiments. Aged mice were then studied over a similar time-frame as adult PDGFRβ-tdTomato mice within the same colony. Both adult and aged mice were maintained within one room at the Seattle Children's Research Institute vivarium.
Blinding	Surgeries were performed and data were collected in an unblinded manner because aged mice were visibly different from adult mice. Analyses were performed blinded to the experimental conditions (e.g. ablation/sham, vessel type, and experimental timepoint).

Reporting for specific materials, systems and methods

We require information from authors about some types of materials, experimental systems and methods used in many studies. Here, indicate whether each material, system or method listed is relevant to your study. If you are not sure if a list item applies to your research, read the appropriate section before selecting a response.

Materials & experimental systems

n/a	Involved in the study	
x		Antibodies
x		Eukaryotic cell lines
x		Palaeontology and archaeology
	x	Animals and other organisms
x		Human research participants
x		Clinical data
x		Dual use research of concern

Methods

n/a	Involved in the study	
x		ChIP-seq
x		Flow cytometry
x		MRI-based neuroimaging

Animals and other organisms

Policy information about studies involving animals; ARRIVE guidelines recommended for reporting animal research

Laboratory animals	Transgenic mice were used for these studies. The core studies were performed on PDGFRβ-tdTomato mice between 3-6 months of age (n=6; 3 female), and 18-24 months of age (n=6; 3 female). For a subset of experiments, we crossed PDGFRβ-tdTomato mice with CX3CR1-GFP or Claudin-eGFP fusion protein mice. PDGFRβ-tdTomato;CX3CR1 mice were aged to 3-6 months (adult, n=5; 2 female) and 18-24 months (aged, n=3; 1 female). PDGFRβ-tdTomato;Claudin-eGFP mice (n=3; 1 female) were aged to 7-12 months. The blood cell flux example (Fig. 7) is from a 19-month-old male PDGFRβ-tdTomato mouse.<br><br>Mice were maintained on a 12-hour light cycle (7:00am on, 7:00pm off). Room temperature and humidity were maintained within 68-79°F (setpoint 73°F) and 30-70% (setpoint 50%), respectively. Mouse chow (LabDiet PicoLab 5053 irradiated diet for standard mice, and LabDiet PicoLab 5058 irradiated diet for breeders) was provided ad libitum.
Wild animals	N/A
Field-collected samples	N/A

Ethics oversight	The Institutional Animal Care and Use Committee at the Seattle Children's Research Institute approved all procedures used in this study (protocol IACUC00419).

Note that full information on the approval of the study protocol must also be provided in the manuscript.
